# Non-ATP-Mimetic Organometallic Protein Kinase Inhibitor

**DOI:** 10.1002/open.201300031

**Published:** 2013-09-05

**Authors:** Kathrin Wähler, Katja Kräling, Holger Steuber, Eric Meggers

**Affiliations:** [a] Fachbereich Chemie, Philipps-Universität Marburg, Hans-Meerwein-Straße35043 Marburg (Germany) E-mail: meggers@chemie.uni-marburg.de; [b] LOEWE-Zentrum für Synthetische Mikrobiologie, Philipps-Universität Marburg, Hans-Meerwein-Straße35043 Marburg (Germany); [d] College of Chemistry and Chemical Engineering, Xiamen UniversityXiamen 361005 (P. R. China)

**Keywords:** bioinorganic chemistry, enzyme inhibitors, organometallic compounds, protein structures, ruthenium

Features such as unusual reactivities, tunable ligand exchange kinetics, structural diversity and complexity, the availability of radioisotopes, and distinct physicochemical properties render organometallics an attractive class of compounds for the development of novel drug candidates and as tools in the life sciences for the modulation, sensing, and imaging of biological processes.[1] For example, over the last several years, substitutionally inert metal complexes have emerged as sophisticated scaffolds for protein targeting.[2] In this respect, Spencer and co-workers reported ferrocene-based inhibitors for the receptor tyrosine kinases VEGFR and EGFR,[3, 4] the dual-specificity kinases DYRK,[5] and histone deacetylases.[6, 7] Poulsen et al. demonstrated the usefulness of metallocenes for the inhibition of carbonic anhydrases.[8, 9] Alberto and co-workers introduced technetium(I) and rhenium(I) half-sandwich complexes as selective inhibitors of human carbonic anhydrase IX,[10] and Ma and co-workers designed cyclometalated iridium(III) and rhodium(III) complexes as inhibitors of the tumor necrosis factor-α[11] and Janus kinase 2,[12] respectively. Our laboratory contributed to this area of research by demonstrating that substitutionally inert ruthenium(II),[13, 14] osmium(II),[15] rhodium(III),[16] iridium(III),[13, 17] and platinum(II)[18] complexes can serve as highly selective and potent ATP-competitive inhibitors for protein kinases and lipid kinases. Our previous design was predominantly based on a staurosporine-inspired metallo-pyridocarbazole scaffold (e.g., Pim1/GSK3 inhibitor **HB12**[19] in Figure 1), in which a maleimide moiety forms one or two key hydrogen bonds with the hinge region of the ATP binding site, the pyridocarbazole heterocycle occupies the hydrophobic adenine binding cleft, and the remaining metal complex fragment can interact within the ribose-triphosphate binding region.[20] Owing the often lengthy synthesis of the pyridocarbazole heterocycle, which also contains one inconvenient photochemical step,[21] we recently became interested in simplifying the design of organometallic protein kinase inhibitors by making use of cyclometalation through C–H activation as a means to reduce the number of required heteroatoms for transition metal binding.[14, 22] Here we now wish to report such a novel cyclometalated protein kinase inhibitor scaffold based on 1,8-phenanthrolin-7(8 *H*)-one cyclometalated with the half-sandwich moiety [Ru(*η*^5^-C_5_H_5_)(CO)] in a bidentate fashion (**1** in Figure 1). Surprisingly, a cocrystal structure of organometallic compound **1** bound to the protein kinase Pim1 reveals an unexpected binding mode in which the amide group of organometallic **1** does not—as initially intended—interact with the hinge region of the ATP binding site, but instead forms hydrogen bonds with the amino acid side chains of Lys67 and Asp186. Thus, the cyclometalated 1,8-phenanthrolin-7(8 *H*)-one **1** may constitute an attractive scaffold for the design of protein kinase inhibitors with novel properties.

**Figure 1:**
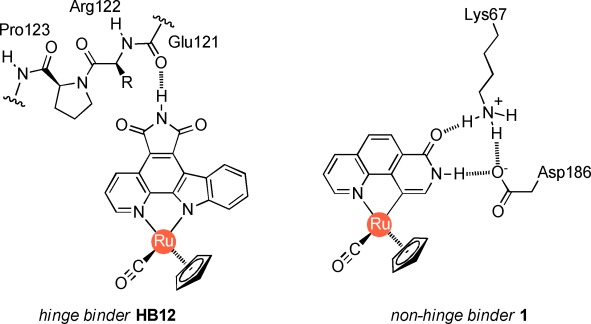
Organometallic protein kinase inhibitors. For the metallo-pyridocarbazole **HB12** (previous design) and metallo-phenanthroline **1** (this study), key interactions to the ATP binding site of Pim1 are shown.

We initiated our study by synthesizing 1,8-phenanthrolin-7(8 *H*)-one (**2**) and its benzylated derivative **2 Bn** according to modified literature procedures (Scheme 1).[23] Accordingly, starting with quinoline-8-carbaldehyde (**3**)[24] a Wittig reaction was used to obtain methyl (2*E*)-3-(quinolin-8-yl)prop-2-enoate (**4**) in a yield of 64 %. Next, reaction of **4** with aqueous sodium hydroxide in methanol afforded carboxylic acid **5**, which was converted to (2*E*)-3-(quinolin-8-yl)prop-2-enoyl azide (**6**) using ethyl chloroformate and sodium azide. In a last step, the desired phenanthroline ligand **2** was obtained via a Curtius rearrangement in 67 % yield. To get the benzylated derivative **2 Bn**, the unprotected ligand was converted using sodium hydride and benzyl bromide in a yield of 98 %. Using the phenanthroline ligands **2** and **2 Bn**, cyclometalations with the ruthenium precursor [Ru(*η*^5^-C_5_H_5_)(CO)(MeCN)_2_]PF_6_[25] in the presence of triethylamine in *N*,*N*-dimethylformamide at 70–80 °C provided the racemic half-sandwich complexes **1** (30 % yield) and **1 Bn** (57 % yield), respectively. Complex **1** was tested to be stable for at least a week on the bench top without exclusion of light and air in the presence of 2-mercaptoethanol (5 mm) in [D_6_]DMSO/D_2_O (9:1 *v*/*v*) as determined by ^1^H NMR spectroscopy (see Supporting Information).

**Scheme 1:**
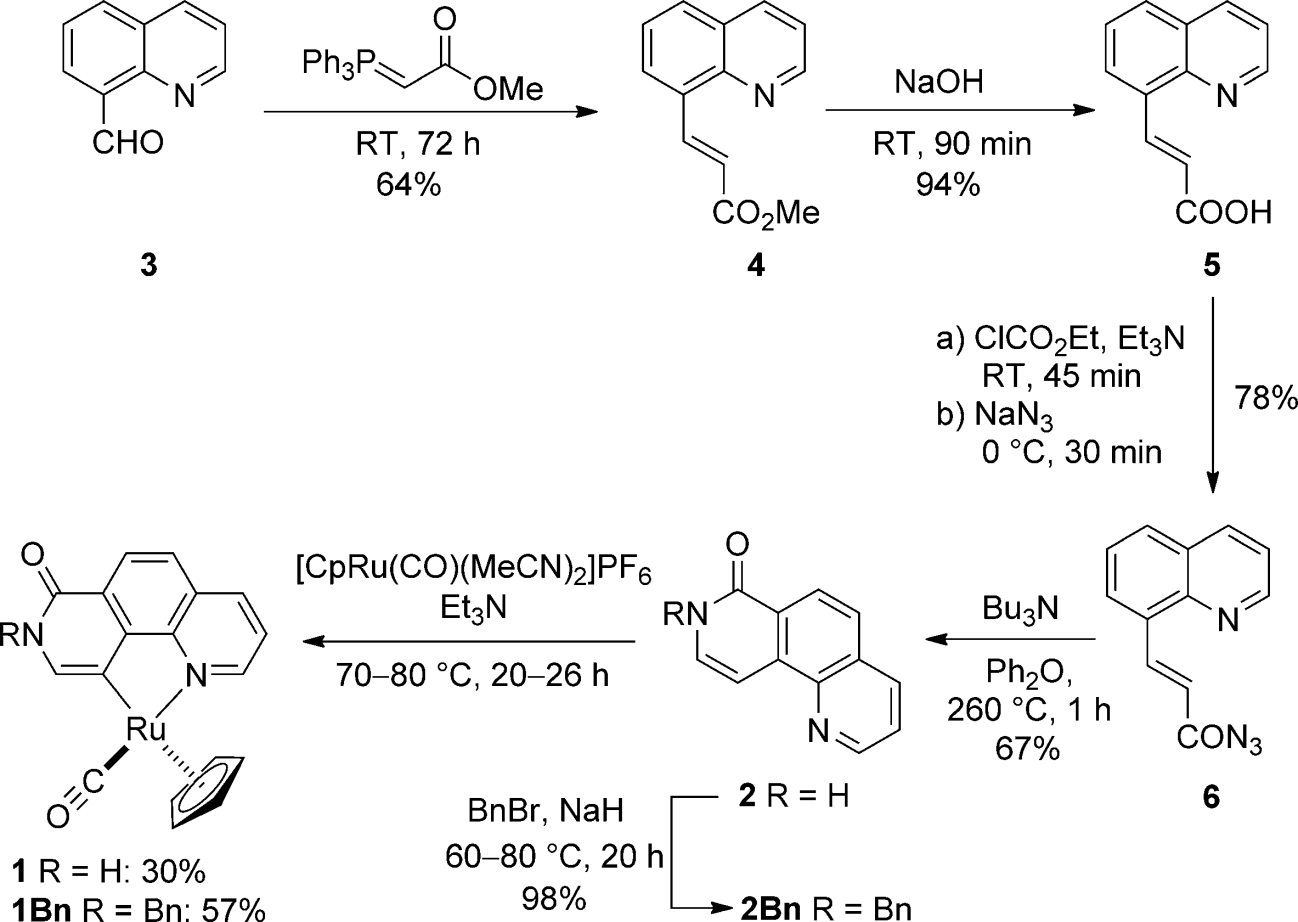
Synthesis of the ruthenium half-sandwich complexes **1** and **1 Bn**.

To gain insight into the protein kinase inhibition properties of this new phenanthroline metal complex scaffold, complex **1** was profiled against the majority of human protein kinases encoded in the human genome (human kinome).[26] This was accomplished by using an active-site-directed competition binding assay with 451 different protein kinases (KINOME*scan*, DiscoveRx), which provides primary data (%ctrl=percent of control: 0 %=highest affinity, 100 %=no affinity) that correlate with dissociation constants (*K*_d_).[27] As a result, out of the tested 451 enzymes, just two protein kinases, namely DYRK1A and Pim2, were identified as the main hits with %ctrl values below 1 % at a concentration of 10 μm**1** (see Supporting Information for more details). Subsequent inhibition assays with organometallic **1** resulted in IC_50_ values of 1.96 μm and 2.09 μm for DYRK1A and Pim2, respectively, at a concentration of 1 μm ATP. As anticipated, the benzylated derivative **1 Bn**, in which the lactam NH group is replaced against *N*-benzyl, does not inhibit protein kinases as verified with an IC_50_ of >100 μm against DYRK1A (1 μm ATP), suggesting that it is not any metal-based reactivity that is responsible for kinase inhibition but instead weak interactions with residues of the metal ligand sphere.

Because of the structural homology of the isoforms Pim1[28–30] and Pim2[31] and our experience with the crystallization of Pim1,[15, 19, 21b, 22b] we cocrystallized **1** with the protein kinase Pim1. The structure of the Pim1/**1** complex was determined and refined to a resolution of 1.95 Å (Table 1). The overall structure shows the typical two-lobe protein kinase architecture connected by a so-called hinge region, and the catalytic ATP binding site positioned in a deep intervening cleft,[28–30] where one enantiomer of the ruthenium complex **1** is located (Figure 2). Unexpectedly, no hydrogen bonds of **1** with the hinge region are observed but instead with amino acid residues, which are located at the opposite site of the active site that is responsible for interacting with the triphosphate unit of ATP directly and mediated through magnesium(II) ions (Figure 3). The amide moiety of organometallic **1** forms a hydrogen bond between the lactam NH of **1** and the carboxylate group of Asp186, an amino acid residue that is part of the DFG loop having the role to bind a magnesium(II) ion during ATP activation.[28] The lactam NH group additionally undergoes a water-mediated contact with the backbone NH group of Asp186. Another hydrogen bond is observed between the carbonyl group of organometallic **1** and the ammonium moiety of the conserved Lys67, an amino acid residue that directly interacts with the α-phosphate of ATP during phospho transfer catalysis.[28] Related non-hinge hydrogen-bonding interactions have been observed with organic Pim1 inhibitors.[29, 30, 32] The phenanthroline ligand as well as the *η*^5^-C_5_H_5_ and the CO ligand of organometallic **1** form a number of hydrophobic contacts, most notably with Leu44, Phe49, Val52, Leu174, and Ile185 (Figure 3). Particularly interesting is the observation that the monodentate CO ligand is directed towards the glycine-rich loop (P-loop) and fills a small hydrophobic pocket formed by induced fit from the residues Leu44, Gly45, Phe49, and Val52 of the flexible glycine-rich loop, thereby being located at a similar position compared to CO ligands in cocrystal structures of related metallo-pyridocarbazole complexes with Pim1 (Figure 4).[15, 19, 21b] It is noteworthy that Pim kinases feature an atypical hinge region due to an insertion of one additional amino acid residue and the presence of a proline, which prevents the formation of a second canonical hydrogen bond with ATP or ATP-mimetic inhibitors.[28–30] It has been suggested by Knapp, Schwaller, and co-workers that non-ATP-mimetic inhibitors which focus on key hydrogen-bonding interactions to other areas of the ATP binding site than the hinge region, such is the case for the here reported organometallic complex **1**, promise to provide an advantage over typical ATP-mimetic inhibitors for achieving high affinities and selectivities for Pim kinases.[32, 33]

**Table 1:** Crystallographic data and refinement statistics for 1/Pim1

PDB code	3WE8
**Data collection and Processing**	
No. of crystals used	1
Wavelength [Å]	0.91841
Space group	*P*6_5_
Unit cell parameters *a*, *b*, *c* [Å] *α*, *β*, *γ* [°]	97.73; 97.73; 81.19 90; 90; 120
Matthews coefficient [Å^3^/Da]	3.1
Solvent content [%]	60.2
Diffraction data	
Resolution range [Å]	50.0–1.95 (2.1–1.95)
Unique reflections	31 990 (6166)
*R*(*I*)_sym_ [%]	10.4 (64.3)
Completeness [%]	100 (100)
Redundancy	7.7 (7.7)
*I*/*σ*(*I*)	14.7 (3.6)
**Refinement**	
Resolution range [Å]	42.32–1.95
Reflections used in refinement (work/free)	31 030/960
Final *R* values for all reflections (work/free) [%]	15.13/17.73
Protein residues	273
Inhibitor	1
Water molecules	285
RMSDs	
Bonds [Å]	0.015
Angles [°]	1.55
**Ramachandran plot**	
Residues in most favoured regions [%]	93.2
Residues in additional allowed regions [%]	6.8
Residues in generously allowed regions [%]	–
**Mean**­ ***B***­ **factor [Å^2^]**	
Protein	29.7
Inhibitor	26.4
Water molecules	40.9

**Figure 2:**
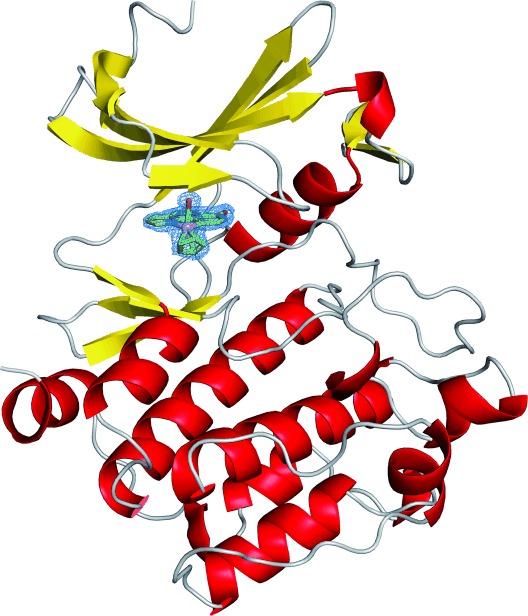
Overview of the crystal structure of Pim1 with one enantiomer of the organoruthenium compound **1** bound to the ATP binding site. The SIGMAA-weighted 2*F*_obs_−*F*_calc_ difference electron density map of the ruthenium inhibitor was contoured at 1*σ*.

**Figure 3:**
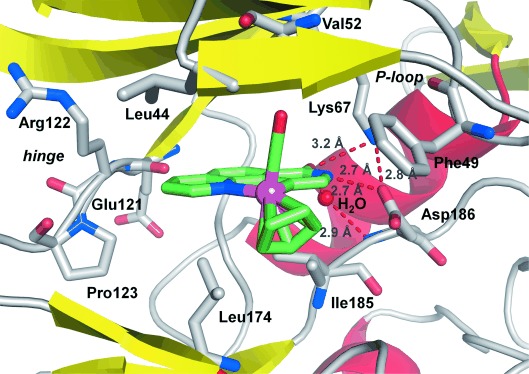
Interactions of **1** within the ATP binding site of Pim1.

**Figure 4:**
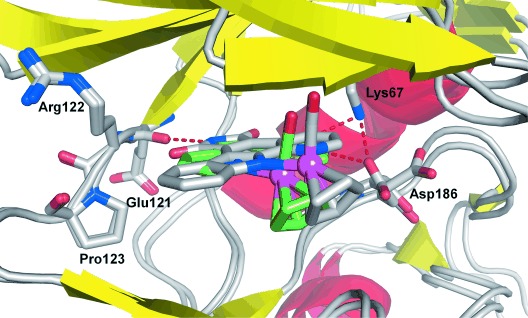
Superimposed cocrystal structures of ruthenium compounds **HB12** (grey) and **1** (green) in the ATP binding site of Pim1. Superimposed with the PyMOL Molecular Graphics System, Version 1.3, Schrödinger, LLC.

In conclusion, we here introduced a new organometallic protein kinase inhibitor scaffold based on a cyclometalated 1,8-phenanthrolin-7(8 *H*)-one ligand. Whereas most kinase inhibitors discovered to date,[34] including all of our previously disclosed metallo-pyridocarbazole complexes, are ATP competitive and present one to three hydrogen bonds to the amino acids located in the hinge region of the target kinase, thereby mimicking the hydrogen-bonding interaction with the adenine nucleobase of ATP, the organometallic compound **1** constitutes an unexpected non-hinge binding scaffold as verified with a Pim1/**1** cocrystal structure, and might constitute a promising lead structure for the development of potent and selective non-hinge-binding ATP-competitive inhibitors of Pim kinases. Pim kinases are interesting targets for cancer therapy as they are overexpressed in various human cancers, associated with metastasis, and overall treatment response.[29, 30]

## Experimental Section

### Synthesis

**Materials and methods:** All reactions were carried out using oven-dried glassware and conducted under a positive pressure of nitrogen. Chemicals were used as received from standard suppliers. Quinoline-8-carbaldehyde[24] and [Ru(*η*^5^-C_5_H_5_)(CO)(MeCN)_2_]PF_6_[25] were prepared according to literature procedures. (2*E*)-3-(Quinolin-8-yl)prop-2-enoyl azide and 1,8-phenanthrolin-7(8 *H*)-one were synthesized according to modified literature procedures.[23] All solvents for chromatography were distilled prior to use. CH_2_Cl_2_ and *N*,*N*-dimethylformamide (DMF) were dried by common methods and freshly distilled prior to use. The high purities of the synthesized compounds were confirmed by ^1^H and ^13^C NMR spectroscopy. NMR spectra were recorded on a DPX-250 (250 MHz), Avance 300 (300 MHz), DRX 400 (400 MHz) or Avance 500 (500 MHz) spectrometer at 298 K. Infrared spectra were recorded on a Bruker Alpha FTIR instrument. High-resolution mass spectra were obtained with a Finnigan LTQ-FT instrument using either APCI or ESI.

**Methyl (2** ***E*****)-3-(quinolin-8-yl)prop-2-enoate (4)**: Quinoline-8-carbaldehyde (1.07 g, 6.81 mmol) and methyl 2-(triphenylphosphoranylidene)acetate (2.73 g, 8.17 mmol) were dissolved in CH_2_Cl_2_ (40 mL), and the solution was stirred for 72 h at 20 °C. The solvent was removed in vacuo. The crude product was subjected to flash silica gel chromatography (4:1 *v*/*v* hexane/EtOAc) to obtain the desired quinoline **4** as a yellow oil (930 mg, 64 %): ^1^H NMR (300 MHz, CDCl_3_): *δ*=8.99 (dd, *J*=4.2, 1.8 Hz, 1 H), 8.93 (d, *J*=16.3 Hz, 1 H), 8.17 (dd, *J*=8.3, 1.8 Hz, 1 H), 7.99 (dd, *J*=7.2, 0.8 Hz, 1 H), 7.87 (dd, *J*=8.2, 1.2 Hz, 1 H), 7.57 (dd, *J*=7.8, 7.6 Hz, 1 H), 7.46 (dd, *J*=8.3, 4.2 Hz, 1 H), 6.83 (d, *J*=16.2 Hz, 1 H), 3.85 ppm (s, 3 H); ^13^C NMR (75 MHz, CDCl_3_): *δ*=170.4, 144.7, 141.7, 138.7, 138.4, 135.3, 129.9, 128.7, 128.2, 125.4, 123.4, 122.7, 45.3 ppm; IR (film): *ṽ*=2948, 1703, 1631, 1494, 1433, 1387, 1311, 1252, 1162, 988, 828, 793, 725 cm^−1^; HRMS (ESI): *m*/*z* [*M*+Na]^+^ calcd for C_13_H_11_NO_2_Na: 236.0682, found: 236.0684.

**(2** ***E*****)-3-(Quinolin-8-yl)prop-2-enoic acid (5)**: Acrylic ester **4** (930 mg, 4.36 mmol) was dissolved in MeOH/6 % NaOH (2:1 *v*/*v*, 18 mL) and stirred for 90 min at 20 °C. The solution was neutralized with 2 m HCl, and the formed precipitate was filtered to give compound **5** as a white solid (821 mg, 94 %): ^1^H NMR (300 MHz, [D_6_]DMSO): *δ*=12.42 (s, 1 H), 9.01 (dd, *J*=4.2, 1.8 Hz, 1 H), 8.84 (d, *J*=16.4 Hz, 1 H), 8.44 (dd, *J*=8.3, 1.8 Hz, 1 H), 8.27 (dd, *J*=7.4, 1.1 Hz, 1 H), 8.09 (dd, *J*=8.2, 1.2 Hz, 1 H), 7.68 (dd, *J*=7.7, 7.7 Hz, 1 H), 7.63 (dd, *J*=8.3, 4.1 Hz, 1 H), 6.84 ppm (d, *J*=16.3 Hz, 1 H); ^13^C NMR (75 MHz, CD_3_CN): *δ*=168.3, 151.5, 146.6, 142.2, 137.6, 131.6, 129.5, 128.9, 127.4, 122.9, 120.4 ppm; IR (film): *ṽ*=2962, 1675, 1615, 1494, 1256, 1016, 789, 598 cm^−1^; HRMS (ESI): *m*/*z* [*M*−H]^−^ calcd for C_12_H_8_NO_2_: 198.0561, found: 198.0557.

**(2** ***E*****)-3-(Quinolin-8-yl)prop-2-enoyl azide (6)**: Acrylic acid **5** (150 mg, 753 μmol) was dissolved in acetone (10 mL). Et_3_N (234 μL, 1.69 mmol) was added, followed by ethyl chloroformate (80 μL, 843 μmol) in acetone (10 mL), and the solution was stirred for 45 min at 20 °C. The mixture was cooled to 0 °C, and a solution of NaN_3_ (93 mg, 1.43 mmol) in H_2_O (4 mL) was added. The mixture was stirred for another 30 min at 0 °C and then poured into ice water. The resulting precipitate was filtered and washed with H_2_O to obtain compound **6** as a white solid (131 mg, 78 %): ^1^H NMR (300 MHz, [D_6_]DMSO): *δ*=9.05–8.98 (m, 2 H), 8.47 (dd, *J*=8.4, 1.7 Hz, 1 H), 8.38 (d, *J*=7.2 Hz, 1 H), 8.15 (d, *J*=8.3 Hz, 1 H), 7.71 (dd, *J*=7.6, 7.3 Hz, 1 H), 7.66 (dd, *J*=8.2, 4.2 Hz, 1 H), 7.06 ppm (d, *J*=16.2 Hz, 1 H). ^13^C NMR (75 MHz, [D_6_]DMSO): *δ*=171.7, 150.9, 145.3, 142.2, 136.8, 131.7, 130.9, 129.1, 128.2, 126.5, 122.2, 120.6 ppm; IR (film): *ṽ*=2144, 2089, 1678, 1614, 1569, 1211, 1172, 823, 789, 694 cm^−1^; HRMS (ESI): *m*/*z* [*M*+H]^+^ calcd for C_12_H_9_N_4_O: 225.0771, found: 225.0772.

**1,8-Phenanthrolin-7(8** ***H*****)-one (2)**: A solution of tributylamine (10.9 mL, 46.0 mmol) in diphenyl ether (65 mL) was added to a solution of azide **6** (860 mg, 3.84 mmol) in diphenyl ether (65 mL). The mixture was stirred for 1 h at 260 °C, allowed to cool to RT and diluted with hexane (300 mL). The resulting precipitate was filtered and washed with hexane to obtain compound **2** as a pale yellow solid (501 mg, 67 %): ^1^H NMR (300 MHz, [D_6_]DMSO): *δ*=11.75 (s, 1 H), 9.06 (dd, *J*=4.4, 1.7 Hz, 1 H), 8.49 (dd, *J*=8.3, 1.7 Hz, 1 H), 8.25 (d, *J*=8.7 Hz, 1 H), 7.95 (d, *J*=8.7 Hz, 1 H), 7.76 (dd, *J*=8.2, 4.3 Hz, 1 H), 7.72 (d, *J*=7.2 Hz, 1 H), 7.51–7.47 ppm (m, 1 H); ^13^C NMR (75 MHz, [D_6_]DMSO): *δ*=161.6, 150.1, 143.3, 137.7, 136.4, 130.9, 129.2, 125.6, 125.4, 123.7, 123.5 ppm; IR (film): *ṽ*=1633, 1588, 1547, 1388, 1237, 918, 839, 771, 701 cm^−1^; HRMS (APCI): *m*/*z* [*M*+H]^+^ calcd for C_12_H_9_N_2_O: 197.0709, found: 197.0710.

**8-Benzyl-1,8-phenanthrolin-7-one (2 Bn)**: A suspension of compound **2** (100 mg, 510 μmol) in *N*,*N*-dimethylformamide (DMF; 2 mL) was cooled to 0 °C, and NaH (60 % mineral oil dispersion, 25 mg, 612 μmol) was added. The mixture was allowed to warm to RT and stirred for 30 min. Benzyl bromide (73 μL, 612 μmol) was added and the mixture was stirred for 4 h at 60 °C and for 16 h at 80 °C. The mixture was cooled to ambient temperature, and the solvent was removed in vacuo. The crude product was subjected to flash silica gel chromatography (3:1 *v*/*v* hexane/EtOAc) to obtain compound **2 Bn** as a white solid (142 mg, 98 %): ^1^H NMR (500 MHz, CDCl_3_): *δ*=9.01 (dd, *J*=4.3, 1.8 Hz, 1 H), 8.50 (d, *J*=8.8 Hz, 1 H), 8.23 (dd, *J*=8.2, 1.7 Hz, 1 H), 7.89 (d, *J*=7.4 Hz, 1 H), 7.81 (d, *J*=8.8 Hz, 1 H), 7.58 (dd, *J*=8.2, 4.2 Hz, 1 H), 7.42 (d, *J*=7.4 Hz, 1 H), 7.38–7.28 (m, 5 H), 5.33 ppm (s, 2 H); ^13^C NMR (125 MHz, CDCl_3_): *δ*=162.2, 149.8, 137.4, 136.8, 136.4, 133.2, 129.8, 129.0, 128.2, 128.1, 126.2, 126.1, 125.1, 123.4, 102.6, 52.4 ppm; IR (film): *ṽ*=1648, 1615, 1593, 1364, 1175, 842, 752, 699 cm^−1^; HRMS (ESI): *m*/*z* [*M*+Na]^+^ calcd for C_19_H_14_N_2_ONa: 309.0998, found: 309.0997.

**Half-sandwich complex 1**: Compound **2** (10 mg, 51 μmol) was dissolved in DMF (2 mL). Et_3_N (19.8 μL, 153 μmol) was added, followed by [Ru(*η*^5^-C_5_H_5_)(CO)(MeCN)_2_]PF_6_ (32 mg, 77 μmol), and the solution was stirred for 26 h at 80 °C. The solution was cooled to ambient temperature, and the solvent was evaporated to dryness in vacuo. The crude product was adsorbed onto silica gel and subjected to flash silica gel chromatography (EtOAc→10:1 *v*/*v* EtOAc/MeOH; 30:1 *v*/*v* CH_2_Cl_2_/MeOH) to obtain the half-sandwich complex **1** as a yellow solid (12 mg, 30 %): ^1^H NMR (300 MHz, [D_6_]DMSO): *δ*=11.45 (s, 1 H), 9.31 (d, *J*=5.1 Hz, 1 H), 8.53 (d, *J*=8.2 Hz, 1 H), 8.12 (d, *J*=8.6 Hz, 1 H), 7.78 (d, *J*=8.6 Hz, 1 H), 7.60 (dd, *J*=8.3, 5.2 Hz, 1 H), 7.11 (d, *J*=5.5 Hz, 1 H), 5.18 ppm (s, 5 H). ^13^C NMR (75 MHz, [D_6_]DMSO): *δ*=205.1, 160.6, 157.8, 153.7, 151.2, 136.4, 134.7, 129.8, 125.9, 125.3, 125.2, 123.6, 122.7, 83.5 ppm: IR (film): *ṽ*=2821, 1904, 1646, 1543, 1464, 1409, 946, 832, 796, 572 cm^−1^; HRMS (ESI): *m*/*z* [*M*+H]^+^ calcd for C_18_H_13_N_2_O_2_Ru: 391.0020, found: 391.0018.

**Half-sandwich complex 1 Bn**: Compound **2 Bn** (10 mg, 35 μmol) was dissolved in DMF (1 mL). Et_3_N (5.8 μL, 42 μmol) was added, followed by [Ru(*η*^5^-C_5_H_5_)(CO)(MeCN)_2_]PF_6_ (24 mg, 101 μmol), and the solution was stirred for 20 h at 70 °C. The solution was cooled to ambient temperature, and the solvent was evaporated to dryness in vacuo. The crude product was subjected to flash silica gel chromatography (1:1 *v*/*v* hexane/EtOAc→EtOAc) to obtain the half-sandwich complex **1 Bn** as a yellow solid (10 mg, 57 %): ^1^H NMR (300 MHz, CD_3_CN): *δ*=9.19 (dd, *J*=5.1, 1.3 Hz, 1 H), 8.36 (dd, *J*=8.2, 1.3 Hz, 1 H), 8.26 (d, *J*=8.7 Hz, 1 H), 7.71 (d, *J*=8.7 Hz, 1 H), 7.48 (dd, *J*=8.3, 5.1 Hz, 1 H), 7.41–7.27 (m, 6 H), 5.39 (d, *J*=14.6 Hz, 1 H), 5.23 (d, *J*=14.6 Hz, 1 H), 5.08 ppm (s, 5 H). ^13^C NMR (125 MHz, CD_3_CN): *δ*=206.0, 161.6, 158.8, 155.4, 151.9, 139.6, 139.2, 137.3, 131.2, 129.6, 128.9, 128.8, 128.4, 126.8, 126.6, 124.5, 124.2, 84.3, 52.7 ppm; IR (film): *ṽ*=1909, 1625, 1553, 1413, 1174, 835, 748, 707, 560, 526 cm^−1^; HRMS (ESI): *m*/*z* [*M*+H]^+^ calcd for C_25_H_19_N_2_O_2_Ru: 481.0491, found: 481.0488.

### Biological evaluation

*Kinase profiling*: The protein kinase selectivity profile of complex **1** at an assay concentration of 10 μm was derived from an active-site-directed affinity screening against 451 human protein kinases (KINOME*scan*, DiscoveRx).[27]

*Protein kinase inhibition assays:* Inhibition data were obtained by a conventional radioactive assay in which DYRK1A (Millipore) and Pim2 (Millipore) activity was measured by the degree of phosphorylation of the respective substrate peptide with [*γ*-^33^P]ATP (PerkinElmer). Accordingly, different concentrations of the ruthenium complexes **1** and **1 Bn** were preincubated at RT for 30 min with the kinase and the substrate peptide (Woodtide peptide substrate (Millipore) for DYRK1A and p70 S6 kinase substrate (Millipore) for Pim2), and the phosphorylation reaction was subsequently initiated by adding ATP and [*γ*-^33^P]ATP. After incubation for 30 min, the reaction was terminated by spotting 25 μL (DYRK1A) or 17.5 μL (Pim2) onto circular P81 phosphocellulose paper (diameter 2.1 cm, Whatman), followed by washing with 0.75 % aq phosphoric acid and acetone. The dried P81 papers were transferred to scintillation vials and scintillation cocktail (5 mL) was added. The counts per minute (CPM) were measured with a Beckmann Coulter LS6500 MultiPurpose Scintillation Counter and corrected by the background CPM. The IC_50_ values were determined in duplicate from sigmoidal curve fits.

DYRK1A inhibition: ATP and [*γ*-^33^P]ATP was added to a final volume of 50 μL, which consisted of Tris-HCl (50 mm, pH 7.5), HEPES (0.5 mm, pH 7.4), Mg(OAc)_2_ (10 mm), DMSO (10 %), DYRK1A (2.2 nm), Woodtide substrate peptide (50 μm), EGTA (0.1 mm), dithiothreitol (15 mm), Brij®-35 (0.03 %), BSA (1.0 mg mL^−1^), and ATP (1.0 μm) including [*γ*-^33^P]ATP (approximately 0.1 μCi μL^−1^).

Pim2 inhibition: ATP and [*γ*-^33^P]ATP was added to a final volume of 25 μL, which consisted of MOPS (10 mm, pH 7.0), Mg(OAc)_2_ (10 mm), DMSO (10 %), Pim2 (15.8 nm), p70 S6 kinase substrate (50 μm), EDTA (0.1 mm), Brij®-35 (0.001 %), glycerol (0.5 %), 2-mercaptoethanol (0.01 %), BSA (0.1 mg mL^−1^), and ATP (1.0 μm) including [*γ*-^33^P]ATP (approximately 0.1 μCi μL^−1^).

### Protein expression, purification, and crystallization

The protein was expressed and purified as described previously.[19] To a solution of Pim1 (8 mg mL^−1^) in HEPES (50 mm, pH 7.5), NaCl (250 mm), DTT (5 mm), and glycerol (5 %) was added the racemic ruthenium complex **1** (10 mm DMSO stock solution) to a concentration of 1 mm, and the mixture was incubated on ice for 1 h. Crystals of nonphosphorylated Pim1 were grown at 4 °C in 4 μL sitting drops, where 2 μL of protein solution were mixed with 2 μL of the precipitation stock containing bis-tris propane (100 mm, pH 7.0), lithium sulfate (200 mm), PEG 3350 (12 %), ethylene glycol (10 %), and DMSO (0.3 %). The final concentration of complex **1** was 0.5 mm and 2.65 % DMSO resulting from the ruthenium stock solution and the precipitation buffer. Crystals were obtained after 3 days and were cryoprotected in the crystallization buffer supplemented with 25 % glycerol before being flash frozen in liquid nitrogen.

### X-Ray crystallography

Data were collected at 100 K using a cryoprotectant solution, consisting of 25 % (*v*/*v*) glycerol in reservoir solution. Raw data were collected at Bessy II (Helmholtz-Zentrum Berlin, Germany), Beamline 14.1.[35] Data processing and scaling was performed using the program XDS.[36] The coordinates of human Pim1 kinase domain as deposited with the Protein Data Bank (PDB) under PDB access code 1XWS were used for molecular replacement via Phaser[37] as implemented in Phenix.[38] Refinement was performed under repeated cycles of manual model building using Coot[39] and crystallographic refinement with the program phenix.refine (version 1.8.1). The final model was validated using PROCHECK.[40] Data collection and refinement statistics are shown in Table 1. The coordinates of the Pim1-ligand complex have been deposited under the PDB accession code 3WE8.

## Supplementary material

As a service to our authors and readers, this journal provides supporting information supplied by the authors. Such materials are peer reviewed and may be re-organized for online delivery, but are not copy-edited or typeset. Technical support issues arising from supporting information (other than missing files) should be addressed to the authors.
