# Supplementary material for: Non-ATP-Mimetic Organometallic Protein Kinase Inhibitor
Source: ChemistryOpen. 2013 Sep 5;2(5-6):180–5. doi: 10.1002/open.201300031 (PMC3892196; doi:10.1002/open.201300031)
Supplement: Supplementary file 1 [file open0002-0180-SD1.pdf]

ChemistryOPEN

Supporting Information

© 2013 The Authors. Published by Wiley-VCH Verlag GmbH & Co. KGaA, Weinheim

Non-ATP-Mimetic Organometallic Protein Kinase Inhibitor

Kathrin Wähler,[a] Katja Kräling,[a] Holger Steuber,*[b, c] and Eric Meggers*[a, d]

open_201300031_sm_miscellaneous_information.pdf

Table of Contents:

I.) Proof of Purity of Complex **1**

II.) Stability Test of Complex **1**

III.) Supplementary Kinase Profiling Data of Complex **1**

I.) Proof of Purity of Complex 1

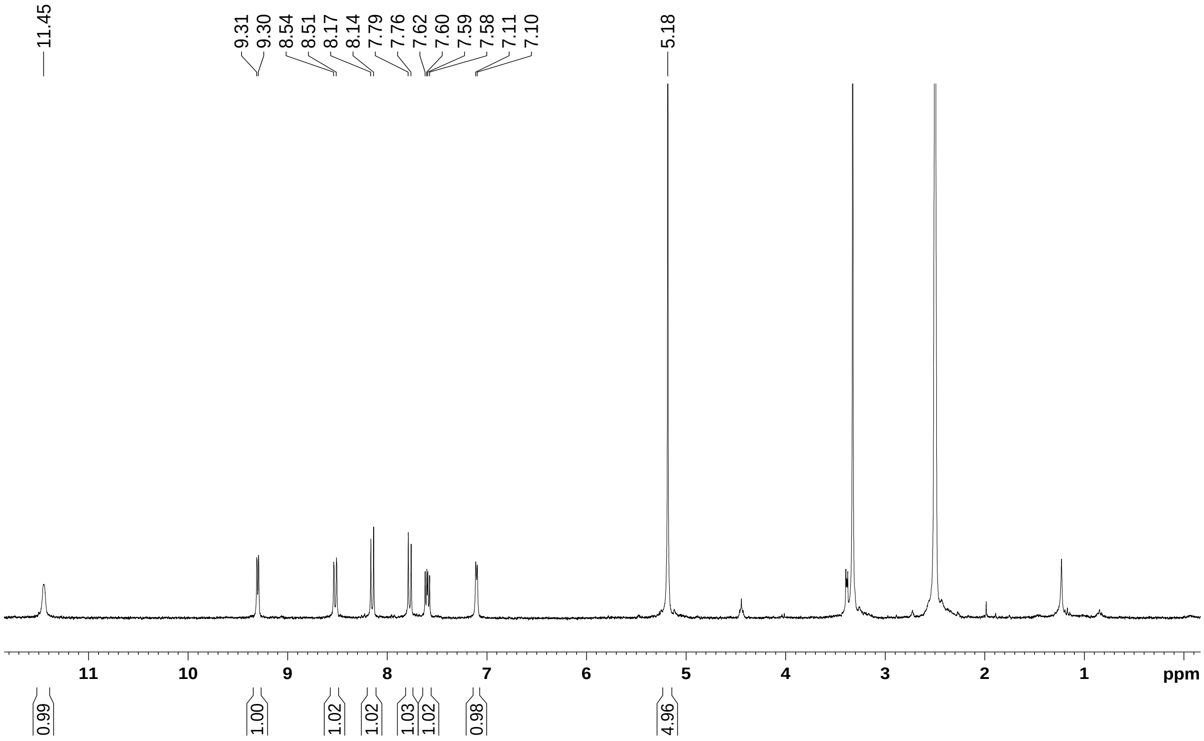

Figure S1. ^{1}H NMR of complex **1** (300 MHz, DMSO-d_6).

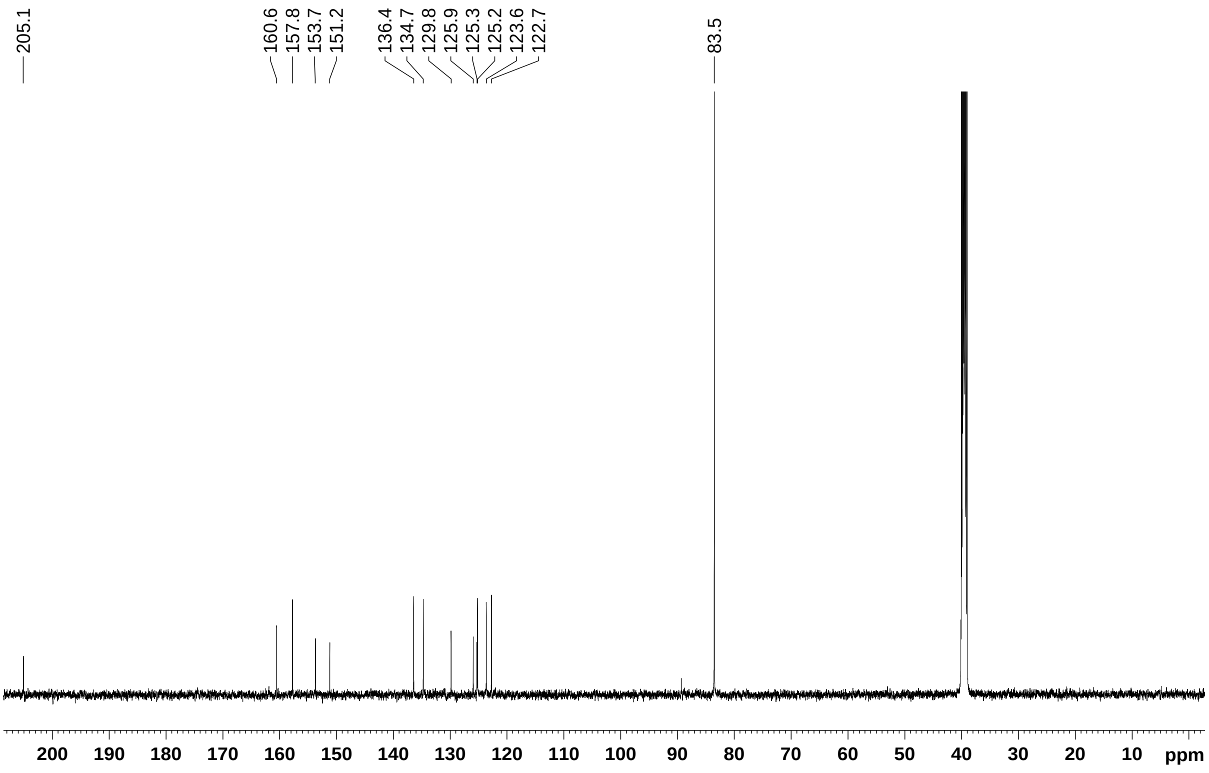

Figure S2. ^{13}C NMR of complex **1** (125 MHz, DMSO-d_6).

II.) Stability Test of Complex 1

To test the stability of the complex under physiological conditions a stock solution (5 mM) in DMSO-d_6/D_2O (9:1, 0.7 mL) of **1** was prepared and 2-mercaptoethanol (5 mM) was added. ^{1}H NMR spectra were measured at the beginning and after 216 h incubation on the benchtop under regular light. Figure S3 shows no change in the spectra after this period of time.

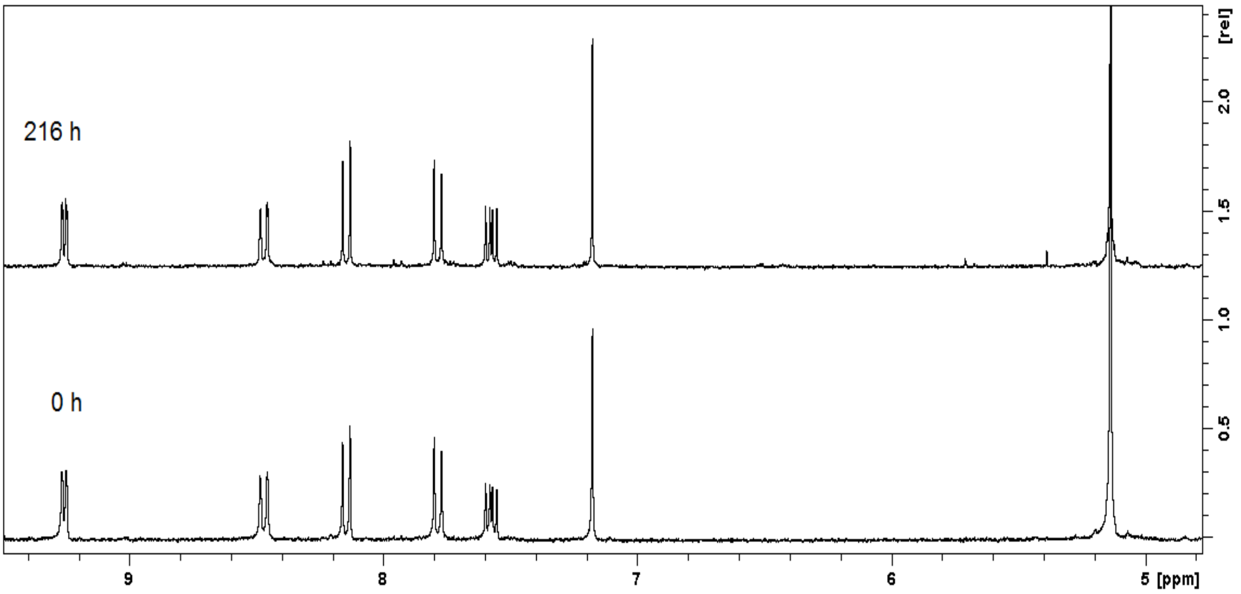

Figure S3. Stability test of complex **1** in addition of 2-mercaptoethanol in DMSO-d_6/D_2O (9:1).

III.) Supplementary Kinase Profiling Data with Complex 1

The protein kinase selectivity profile of racemic complex **1** at an assay concentration of 10 μM was derived from an active-site-directed affinity screening against 451 human protein kinases (KINOME*scan*, DiscoveRx).

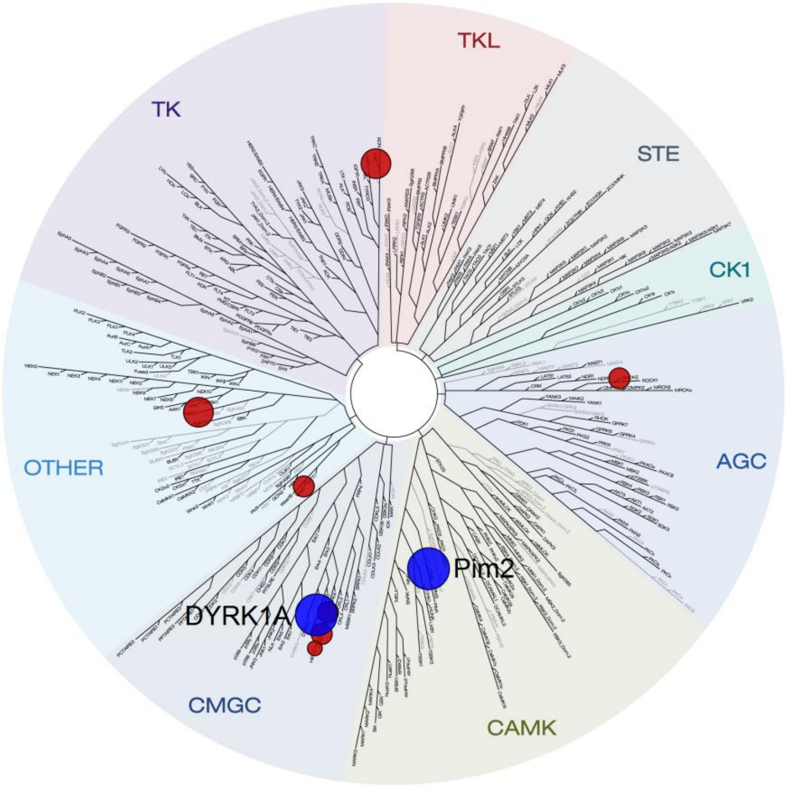

Figure S4. Protein kinase selectivity of complex **1** (10 μM) as determined by an active-site-directed affinity screening (KINOMEscan, DiscoveRx) against 451 human protein kinases. Representation of the main hits (< 1% of control, large blue circles; < 10% of control, red circles) within the human kinase dendrogram which displays the protein kinase families and the evolutionary relationships between the individual kinases. Pim2 – 0.55 %, DYRK1A – 0.8 %.

Table S1. KINOME*scan* Screening Data for Complex **1** (DiscoveRx).

Ambit Gene Symbol	Entrez Gene Symbol	Percent Control
AAK1	AAK1	11
ABL1(E255K)-phosphorylated	ABL1	100
ABL1(F317I)-nonphosphorylated	ABL1	100
ABL1(F317I)-phosphorylated	ABL1	85
ABL1(F317L)-nonphosphorylated	ABL1	100
ABL1(F317L)-phosphorylated	ABL1	89
ABL1(H396P)-nonphosphorylated	ABL1	75
ABL1(H396P)-phosphorylated	ABL1	100
ABL1(M351T)-phosphorylated	ABL1	81
ABL1(Q252H)-nonphosphorylated	ABL1	96
ABL1(Q252H)-phosphorylated	ABL1	100
ABL1(T315I)-nonphosphorylated	ABL1	100
ABL1(T315I)-phosphorylated	ABL1	94
ABL1(Y253F)-phosphorylated	ABL1	100
ABL1-nonphosphorylated	ABL1	92
ABL1-phosphorylated	ABL1	100
ABL2	ABL2	96
ACVR1	ACVR1	100
ACVR1B	ACVR1B	100
ACVR2A	ACVR2A	98
ACVR2B	ACVR2B	81
ACVRL1	ACVRL1	100
ADCK3	CABC1	93
ADCK4	ADCK4	100
AKT1	AKT1	89
AKT2	AKT2	86
AKT3	AKT3	81
ALK	ALK	60
AMPK-alpha1	PRKAA1	92
AMPK-alpha2	PRKAA2	97
ANKK1	ANKK1	86
ARK5	NUAK1	93
ASK1	MAP3K5	62
ASK2	MAP3K6	91
AURKA	AURKA	99
AURKB	AURKB	71
AURKC	AURKC	33
AXL	AXL	51
BIKE	BMP2K	17
BLK	BLK	81
BMPR1A	BMPR1A	100
BMPR1B	BMPR1B	50
BMPR2	BMPR2	39

BMX	BMX	92
BRAF	BRAF	75
BRAF(V600E)	BRAF	86
BRK	PTK6	80
BRSK1	BRSK1	100
BRSK2	BRSK2	100
BTK	BTK	95
BUB1	BUB1	100
CAMK1	CAMK1	99
CAMK1D	CAMK1D	93
CAMK1G	CAMK1G	49
CAMK2A	CAMK2A	16
CAMK2B	CAMK2B	25
CAMK2D	CAMK2D	37
CAMK2G	CAMK2G	46
CAMK4	CAMK4	100
CAMKK1	CAMKK1	49
CAMKK2	CAMKK2	54
CASK	CASK	43
CDC2L1	CDK11B	100
CDC2L2	CDC2L2	100
CDC2L5	CDK13	86
CDK11	CDK19	100
CDK2	CDK2	100
CDK3	CDK3	83
CDK4-cyclinD1	CDK4	45
CDK4-cyclinD3	CDK4	91
CDK5	CDK5	77
CDK7	CDK7	91
CDK8	CDK8	100
CDK9	CDK9	92
CDKL1	CDKL1	92
CDKL2	CDKL2	100
CDKL3	CDKL3	82
CDKL5	CDKL5	78
CHEK1	CHEK1	88
CHEK2	CHEK2	83
CIT	CIT	56
CLK1	CLK1	41
CLK2	CLK2	21
CLK3	CLK3	42
CLK4	CLK4	31
CSF1R	CSF1R	87
CSF1R-autoinhibited	CSF1R	70
CSK	CSK	100
CSNK1A1	CSNK1A1	79
CSNK1A1L	CSNK1A1L	99

CSNK1D	CSNK1D	63
CSNK1E	CSNK1E	68
CSNK1G1	CSNK1G1	81
CSNK1G2	CSNK1G2	100
CSNK1G3	CSNK1G3	100
CSNK2A1	CSNK2A1	100
CSNK2A2	CSNK2A2	51
CTK	MATK	100
DAPK1	DAPK1	80
DAPK2	DAPK2	45
DAPK3	DAPK3	62
DCAMKL1	DCLK1	91
DCAMKL2	DCLK2	70
DCAMKL3	DCLK3	99
DDR1	DDR1	96
DDR2	DDR2	100
DLK	MAP3K12	97
DMPK	DMPK	68
DMPK2	CDC42BPG	86
DRAK1	STK17A	64
DRAK2	STK17B	87
DYRK1A	DYRK1A	0.8
DYRK1B	DYRK1B	25
DYRK2	DYRK2	18
EGFR	EGFR	89
EGFR(E746-A750del)	EGFR	100
EGFR(G719C)	EGFR	100
EGFR(G719S)	EGFR	100
EGFR(L747-E749del, A750P)	EGFR	77
EGFR(L747-S752del, P753S)	EGFR	100
EGFR(L747-T751del,Sins)	EGFR	85
EGFR(L858R)	EGFR	98
EGFR(L858R,T790M)	EGFR	89
EGFR(L861Q)	EGFR	87
EGFR(S752-I759del)	EGFR	100
EGFR(T790M)	EGFR	45
EIF2AK1	EIF2AK1	100
EPHA1	EPHA1	88
EPHA2	EPHA2	99
EPHA3	EPHA3	78
EPHA4	EPHA4	100
EPHA5	EPHA5	84
EPHA6	EPHA6	77
EPHA7	EPHA7	63
EPHA8	EPHA8	50
EPHB1	EPHB1	84
EPHB2	EPHB2	84

EPHB3	EPHB3	86
EPHB4	EPHB4	94
EPHB6	EPHB6	35
ERBB2	ERBB2	100
ERBB3	ERBB3	100
ERBB4	ERBB4	100
ERK1	MAPK3	90
ERK2	MAPK1	92
ERK3	MAPK6	85
ERK4	MAPK4	81
ERK5	MAPK7	100
ERK8	MAPK15	42
ERN1	ERN1	96
FAK	PTK2	100
FER	FER	100
FES	FES	72
FGFR1	FGFR1	93
FGFR2	FGFR2	100
FGFR3	FGFR3	74
FGFR3(G697C)	FGFR3	67
FGFR4	FGFR4	100
FGR	FGR	86
FLT1	FLT1	73
FLT3	FLT3	15
FLT3(D835H)	FLT3	35
FLT3(D835Y)	FLT3	68
FLT3(ITD)	FLT3	46
FLT3(K663Q)	FLT3	21
FLT3(N841I)	FLT3	56
FLT3(R834Q)	FLT3	74
FLT3-autoinhibited	FLT3	61
FLT4	FLT4	89
FRK	FRK	100
FYN	FYN	100
GAK	GAK	69
GCN2(Kin.Dom.2,S808G)	EIF2AK4	77
GRK1	GRK1	75
GRK4	GRK4	32
GRK7	GRK7	72
GSK3A	GSK3A	47
GSK3B	GSK3B	59
HASPIN	GSG2	4
HCK	HCK	100
HIPK1	HIPK1	7.7
HIPK2	HIPK2	10
HIPK3	HIPK3	8
HIPK4	HIPK4	23

HPK1	MAP4K1	93
HUNK	HUNK	77
ICK	ICK	100
IGF1R	IGF1R	99
IKK-alpha	CHUK	100
IKK-beta	IKBKB	100
IKK-epsilon	IKBKE	100
INSR	INSR	61
INSRR	INSRR	81
IRAK1	IRAK1	73
IRAK3	IRAK3	61
IRAK4	IRAK4	99
ITK	ITK	89
JAK1(JH1domain-catalytic)	JAK1	100
JAK1(JH2domain-pseudokinase)	JAK1	17
JAK2(JH1domain-catalytic)	JAK2	88
JAK3(JH1domain-catalytic)	JAK3	29
JNK1	MAPK8	46
JNK2	MAPK9	75
JNK3	MAPK10	50
KIT	KIT	62
KIT(A829P)	KIT	100
KIT(D816H)	KIT	85
KIT(D816V)	KIT	60
KIT(L576P)	KIT	62
KIT(V559D)	KIT	49
KIT(V559D,T670I)	KIT	96
KIT(V559D,V654A)	KIT	89
KIT-autoinhibited	KIT	84
LATS1	LATS1	81
LATS2	LATS2	33
LCK	LCK	98
LIMK1	LIMK1	95
LIMK2	LIMK2	93
LKB1	STK11	85
LOK	STK10	84
LRRK2	LRRK2	59
LRRK2(G2019S)	LRRK2	96
LTK	LTK	87
LYN	LYN	87
LZK	MAP3K13	100
MAK	MAK	100
MAP3K1	MAP3K1	74
MAP3K15	MAP3K15	100
MAP3K2	MAP3K2	62
MAP3K3	MAP3K3	76
MAP3K4	MAP3K4	100

MAP4K2	MAP4K2	64
MAP4K3	MAP4K3	78
MAP4K4	MAP4K4	93
MAP4K5	MAP4K5	99
MAPKAPK2	MAPKAPK2	100
MAPKAPK5	MAPKAPK5	100
MARK1	MARK1	91
MARK2	MARK2	83
MARK3	MARK3	67
MARK4	MARK4	90
MAST1	MAST1	100
MEK1	MAP2K1	77
MEK2	MAP2K2	93
MEK3	MAP2K3	100
MEK4	MAP2K4	57
MEK5	MAP2K5	100
MEK6	MAP2K6	100
MELK	MELK	56
MERTK	MERTK	71
MET	MET	4.8
MET(M1250T)	MET	23
MET(Y1235D)	MET	40
MINK	MINK1	86
MKK7	MAP2K7	100
MKNK1	MKNK1	100
MKNK2	MKNK2	24
MLCK	MYLK3	65
MLK1	MAP3K9	70
MLK2	MAP3K10	100
MLK3	MAP3K11	100
MRCKA	CDC42BPA	98
MRCKB	CDC42BPB	41
MST1	STK4	91
MST1R	MST1R	73
MST2	STK3	61
MST3	STK24	65
MST4	MST4	92
MTOR	MTOR	100
MUSK	MUSK	90
MYLK	MYLK	54
MYLK2	MYLK2	100
MYLK4	MYLK4	26
MYO3A	MYO3A	86
MYO3B	MYO3B	100
NDR1	STK38	100
NDR2	STK38L	60
NEK1	NEK1	100

NEK11	NEK11	100
NEK2	NEK2	100
NEK3	NEK3	100
NEK4	NEK4	100
NEK5	NEK5	73
NEK6	NEK6	53
NEK7	NEK7	82
NEK9	NEK9	97
NIM1	MGC42105	100
NLK	NLK	82
OSR1	OXSR1	61
p38-alpha	MAPK14	97
p38-beta	MAPK11	100
p38-delta	MAPK13	64
p38-gamma	MAPK12	100
PAK1	PAK1	100
PAK2	PAK2	82
PAK3	PAK3	87
PAK4	PAK4	97
PAK6	PAK6	95
PAK7	PAK7	100
PCTK1	CDK16	86
PCTK2	CDK17	100
PCTK3	CDK18	67
PDGFRA	PDGFRA	93
PDGFRB	PDGFRB	71
PDPK1	PDPK1	100
PFCDPK1(P.falciparum)	CDPK1	100
PFPK5(P.falciparum)	MAL13P1.279	100
PFTAIRE2	CDK15	98
PFTK1	CDK14	73
PHKG1	PHKG1	100
PHKG2	PHKG2	42
PIK3C2B	PIK3C2B	95
PIK3C2G	PIK3C2G	84
PIK3CA	PIK3CA	96
PIK3CA(C420R)	PIK3CA	80
PIK3CA(E542K)	PIK3CA	100
PIK3CA(E545A)	PIK3CA	100
PIK3CA(E545K)	PIK3CA	100
PIK3CA(H1047L)	PIK3CA	100
PIK3CA(H1047Y)	PIK3CA	76
PIK3CA(I800L)	PIK3CA	90
PIK3CA(M1043I)	PIK3CA	100
PIK3CA(Q546K)	PIK3CA	86
PIK3CB	PIK3CB	100
PIK3CD	PIK3CD	86

PIK3CG	PIK3CG	62
PIK4CB	PI4KB	100
PIM1	PIM1	39
PIM2	PIM2	0.55
PIM3	PIM3	19
PIP5K1A	PIP5K1A	58
PIP5K1C	PIP5K1C	52
PIP5K2B	PIP4K2B	28
PIP5K2C	PIP4K2C	100
PKAC-alpha	PRKACA	96
PKAC-beta	PRKACB	74
PKMYT1	PKMYT1	75
PKN1	PKN1	100
PKN2	PKN2	56
PKNB(M.tuberculosis)	pknB	67
PLK1	PLK1	96
PLK2	PLK2	85
PLK3	PLK3	100
PLK4	PLK4	53
PRKCD	PRKCD	100
PRKCE	PRKCE	76
PRKCH	PRKCH	63
PRKCI	PRKCI	100
PRKCQ	PRKCQ	100
PRKD1	PRKD1	100
PRKD2	PRKD2	100
PRKD3	PRKD3	84
PRKG1	PRKG1	77
PRKG2	PRKG2	58
PRKR	EIF2AK2	82
PRKX	PRKX	100
PRP4	PRPF4B	61
PYK2	PTK2B	93
QSK	KIAA0999	100
RAF1	RAF1	100
RET	RET	92
RET(M918T)	RET	61
RET(V804L)	RET	72
RET(V804M)	RET	100
RIOK1	RIOK1	36
RIOK2	RIOK2	70
RIOK3	RIOK3	8.2
RIPK1	RIPK1	82
RIPK2	RIPK2	100
RIPK4	RIPK4	100
RIPK5	DSTYK	79
ROCK1	ROCK1	55

ROCK2	ROCK2	9.4
ROS1	ROS1	97
RPS6KA4(Kin.Dom.1-N-terminal)	RPS6KA4	83
RPS6KA4(Kin.Dom.2-C-terminal)	RPS6KA4	65
RPS6KA5(Kin.Dom.1-N-terminal)	RPS6KA5	100
RPS6KA5(Kin.Dom.2-C-terminal)	RPS6KA5	43
RSK1(Kin.Dom.1-N-terminal)	RPS6KA1	100
RSK1(Kin.Dom.2-C-terminal)	RPS6KA1	51
RSK2(Kin.Dom.1-N-terminal)	RPS6KA3	59
RSK2(Kin.Dom.2-C-terminal)	RPS6KA3	100
RSK3(Kin.Dom.1-N-terminal)	RPS6KA2	100
RSK3(Kin.Dom.2-C-terminal)	RPS6KA2	75
RSK4(Kin.Dom.1-N-terminal)	RPS6KA6	41
RSK4(Kin.Dom.2-C-terminal)	RPS6KA6	66
S6K1	RPS6KB1	90
SBK1	SBK1	81
SGK	SGK1	87
SgK110	SgK110	100
SGK3	SGK3	54
SIK	SIK1	80
SIK2	SIK2	100
SLK	SLK	96
SNARK	NUAK2	37
SNRK	SNRK	100
SRC	SRC	84
SRMS	SRMS	52
SRPK1	SRPK1	26
SRPK2	SRPK2	100
SRPK3	SRPK3	31
STK16	STK16	84
STK33	STK33	68
STK35	STK35	87
STK36	STK36	72
STK39	STK39	80
SYK	SYK	100
TAK1	MAP3K7	33
TAOK1	TAOK1	40
TAOK2	TAOK2	100
TAOK3	TAOK3	74
TBK1	TBK1	100
TEC	TEC	83
TESK1	TESK1	76
TGFBR1	TGFBR1	91
TGFBR2	TGFBR2	98
TIE1	TIE1	88
TIE2	TEK	100
TLK1	TLK1	78

TLK2	TLK2	73
TNIK	TNIK	84
TNK1	TNK1	84
TNK2	TNK2	96
TNNI3K	TNNI3K	84
TRKA	NTRK1	33
TRKB	NTRK2	20
TRKC	NTRK3	41
TRPM6	TRPM6	100
TSSK1B	TSSK1B	100
TTK	TTK	50
TXK	TXK	100
TYK2(JH1domain-catalytic)	TYK2	55
TYK2(JH2domain-pseudokinase)	TYK2	100
TYRO3	TYRO3	90
ULK1	ULK1	39
ULK2	ULK2	100
ULK3	ULK3	53
VEGFR2	KDR	100
VRK2	VRK2	89
WEE1	WEE1	7.2
WEE2	WEE2	20
WNK1	WNK1	100
WNK3	WNK3	91
YANK1	STK32A	93
YANK2	STK32B	89
YANK3	STK32C	74
YES	YES1	100
YSK1	STK25	99
YSK4	YSK4	23
ZAK	ZAK	85
ZAP70	ZAP70	81
